# Effect of bevacizumab combined with chemotherapy on SDF-1 and CXCR4 in epithelial ovarian cancer and its prognosis

**DOI:** 10.1186/s12957-022-02621-2

**Published:** 2022-05-11

**Authors:** Chunyan Ma

**Affiliations:** Department of Gynecology, the Third People’s Hospital of Jinan City, No.1 Wangsheren North Street, Gongye North Road, Licheng District, Jinan, 250132 Shandong Province China

**Keywords:** Bevacizumab, Chemotherapy, Epithelial ovarian cancer, SDF-1, CXCR4

## Abstract

**Background:**

The effect of bevacizumab combined with chemotherapy on the expression of stromal cell-derived factor-1 (SDF-1) and receptor CXCR4 in epithelial ovarian cancer tumor cells and its prognosis are unknown. Our work aimed to investigate the effect of chemotherapy +/− bevacizumab on these markers and the impact of this treatment modality in clinical outcomes.

**Methods:**

Altogether 68 patients with epithelial ovarian cancer who were treated with chemotherapy in our hospital from June 2018 to June 2019 were selected. It was an open-labeled and controlled clinical trial (ethical approval no. 20180435). The patients were grouped according to their admission order. Patients treated with paclitaxel and carboplatin were included in group A, while patients treated with bevacizumab, paclitaxel, and carboplatin were included in group B. qRT-PCR was used to detect the changes of SDF-1 and CXCR4 before and after chemotherapy. Various clinical indicators of patients in the two groups were recorded to analyze the clinical efficacy, and safety of different treatment modalities and the prognosis of the two groups was analyzed.

**Results:**

The relative expression of SDF-1 and CXCR4 was positively correlated with epithelial ovarian cancer stages (*P*<0.00). Together, SDF-1 and CXCR4 were positively correlated in epithelial ovarian cancer staging (*P*<0.001). SDF-1 and CXCR4 in both groups after chemotherapy were significantly decreased (*P*<0.001), and the downregulation of SDF-1 and CXCR4 expression in group B was significantly higher than that in group A after chemotherapy (*P*<0.001). No significant difference in the metastasis rates of the two groups before chemotherapy was observed (*P*>0.05), but the recurrence rate after 1 year was lower in group B than in group A (*P*<0.05).

**Conclusion:**

Adding bevacizumab diminished the expression of related cancer markers SDF-1 and CXCR4 more than chemotherapy alone in patients with epithelial ovarian cancer. Furthermore, better rates of recurrence with no concerns regarding adverse drug reactions or quality of life were seen in bevacizumab plus chemotherapy group.

## Background

Ovarian cancer has many pathological types, of which epithelial ovarian cancer is an important cause of death from gynecologic malignancies. Epithelial ovarian cancer is an aggressive disease, and currently, it is generally accepted that the disease has a diverse histological classification [1, 2]. The current standardized treatment regimen for this neoplastic disease is based on surgical treatment, supplemented by chemotherapy [3]. At present, chemotherapy is mostly based on platinum drugs, which have a significant effect in delaying disease progression and prolonging patient survival [4, 5]. Among them, carboplatin is relatively common, which is able to inhibit tumor cell generation by binding DNA molecules, and paclitaxel can stabilize and enhance the polymerization of tubulin, prevent depolymerization of microtubules, and inhibit cell mitosis, thereby exerting anti-tumor effects [6, 7]. Some studies have confirmed that the progression of ovarian cancer disease is the result of multiple mechanisms, the most important of which is angiogenesis, a complex pathological and physiological process regulated by a larger number of positive and negative regulators, while vascular endothelial cell growth factor (VEGF) is the most specific and effective one, thus inhibiting VEGF may decrease angiogenesis and slow the progression of disease in ovarian cancer patients. Bevacizumab is a monoclonal antibody drug that inhibits vascular growth; its mechanism of action is to block VEGF induced proliferation of vascular endothelial cells and the production of corresponding cytokines, thus inhibiting tumor angiogenesis [8–10]. The chemokine stromal cell derived factor-1 (SDF-1) interacts with its only receptor CXCR4 and plays an important role in the biological behavior of cancer cells [11–13]. CXCR4 is found in a variety of human cancers, such as breast cancer, prostate cancer, and oral squamous cell carcinoma, and CXCR4 expression is also found in ovarian cancer tissues and cell strains [14, 15]. Studies in recent years have confirmed the close link between SDF-1/CXCR4 axis and tumor cell proliferation, infiltration, and directional migration. In this study, a clinical trial was carried out to analyze the effect of bevacizumab combined with chemotherapy on SDF-1 and CXCR4 in epithelial ovarian cancer patients and specify the relationship between the combined modality and occurrence and progression of tumor, so as to provide a new idea for treatment and prognosis of patients.

## Methods

### General data

Altogether 68 patients with epithelial ovarian cancer who were treated with chemotherapy in our hospital from June 2018 to June 2019 were selected. It was an open-labeled and controlled clinical trial (ethical approval no. 20180435). The patients were grouped according to their admission order. Patients treated with the combination of paclitaxel and carboplatin were included in group A, while patients treated with the combination of bevacizumab, paclitaxel, and carboplatin were included in group B. All patients included met the International Diagnostic Criteria for Epithelial Ovarian Cancer [16] were verified by pathological examination, received surgical treatment, and had no recurrent ovarian cancer. Patients were excluded if they were resistant to platinum drugs and had neurological diseases, liver, renal, or severe organ dysfunction; if serious complications occurred during the treatment, it will result in inability to continue treatment and to complete follow-up. Patients and their families all signed informed consent forms in advance.

### Treatment methods [17]

Treatment regimens for group A and group B were given as described below:

- In Group A, paclitaxel 175 mg/m2 IV over 3 h and carboplatin AUC 5-6 mg·min/mL IV were given over 1 h at day 1. It was repeated every 3 weeks for 6 cycles.
- In Group B, paclitaxel 175 mg/m2 IV over 3 h and carboplatin AUC 6 mg·min/mL IV were given over 1 h at day 1. It was repeated every 3 weeks for 6 cycles. On day 1 of cycle 2, bevacizumab 15 mg/kg IV was given over 30–90 min, which were given every 3 weeks for up to 22 cycles.

### Evaluation criteria

Follow-up observation was carried out to the patients for efficacy evaluation. According to the unified criteria for measurable or evaluable lesions of the World Health Organization (WHO) [18], the efficacy was classified as complete response (CR, complete disappearance of tumor for over 4 weeks), partial response (PR, ≥ decrease of tumor for over 4 weeks, no new lesions), stable disease (SD, increase or decrease of tumor less than 50%), and progressive disease (PD, > 50% increase of tumor, new lesions). The total effective rate was defined as patients with CR plus PR and divided by the total number of cases. The quality of life of the patients after chemotherapy was evaluated by adopting the Quality of Life - Cancer (QOL-C30) scale [19], with the scoring items including physical health, mental health, material life and social function, and the total score of each item was 100 points. The score was positively related to the quality of life, that is the high score indicated the high the quality of life of the patient.

### Outcome measures

The general clinical data, QOL-C30 scale scores, clinical effects, and the adverse reactions of the two groups of patients with epithelial ovarian cancer were compared. qRT-PCR was used to detect the changes of SDF-1 and CXCR4 before and after chemotherapy. The prognosis of the two groups was analyzed.

### qRT-PCR detection of SDF-1 and CXCR4

All patients received the cytoreductive surgery for the first time. After resection, a part of the tissues was put into the 10% formalin (manufacturer: Taiyuan Jiaquan Chemical Co., Ltd.) immediately to fix for 4 h and then embedded in paraffin for serial slices (4-μm thick), and another part of the tissues was put into the liquid nitrogen tank quickly and stored in −80 °C refrigerator. The qRT-PCR technique was used to detect mRNA expression in tissues with the kits purchased from Guangzhou RiboBio Co., Ltd. The total RNA of tissues was extracted according to the operation instructions on the kits and dissolved in 20 μL of DEPC-treated water (manufacturer: Shanghai Shifeng Biological Technology Co., Ltd.). Then, reverse transcription was carried out on the total RNA by using a reverse transcription kit (manufacturer: Beijing Baiaolaibo Technology Co., Ltd.), and the synthesized c DNA was used as a template to carry out qRT-PCR amplification. Reaction conditions: pre-denaturation 95 °C for 15 min, denaturation at 95 °C for 15 s; annealing at 58 °C for 30 s, for a total of 35 cycles; and finally, extension at 72 °C for 15 min. Each sample was provided with 3 multiple wells for 3 repeated tests. After the reaction was completed, the amplification curve and melting curve of real-time PCR were confirmed, and the relative amount of the target gene was calculated according to the result parameters. Relative quantification of target genes was calculated by 2^-△ CT^ (Table 1).

**Table 1:** SDF-1, CXCR4, and their internal reference primer sequences

Genes	Forward primer	Reverse primer
SDF-1 [20]	5′-CCCGAAGCTAAAGTGGATTC-3′	5′-TTCAGAGCTGGGCTCCTACT-3′
CXCR4 [21]	5′-GGCCCTCAAGACCACAGTC-3′	5′-TTAGCTGGAGTGAAAACTTG-3′
GAPDH [21]	5′-CAAAGGTGGATCAGATTCAAG-3′	5′-GGTGAGCATTATCACCCAGAA-3′

### Statistical methods

SPSS 17.0 (Beijing Strong-Vinda Information Technology Co., Ltd.) was applied for statistical analysis. The counting data were represented by [*n*(%)], and the comparison between the observation group and the control group was performed by *X*^2^ test. The measurement data were expressed by (*x*±*s*), i.e., mean value plus/minus standard deviation. The comparison between group A and group B was conducted by independent sample *t* test, and paired *t* test was used before and after nursing. When P<0.05, the difference was statistically significant.

## Results

### General clinical data of two groups of patients

There was no statistical significance in age, smoking history, differentiation, lymph node metastasis, and staging of epithelial ovarian cancer, but the family history of epithelial ovarian cancer had a statistical significance (*P*>0.05, Table 2).

**Table 2:** General clinical data of two groups of patients

Group	Group A (*n*=34)	Group B (*n*=34)	P
Age (years)			
≤61.00	5(14.71)	3(8.82)	0.452
>61.00	29(85.29)	31(91.18)	
Weight (kg)	58.90±8.15	57.70±9.00	0.566
Residence			
Rural	26(76.47)	23(67.65)	0.418
Urban	8(23.53)	11(32.35)	
Family history of epithelial ovarian cancer			
Yes	0(0.00)	4(11.76)	0.039
No	34(100.00)	30(88.24)	
Smoking history			
Yes	32(94.12)	30(88.24)	0.393
No	2(5.88)	4(11.76)	
Differentiation			
High	0(0.00)	0(0.00)	0.604
Medium	10(29.41)	12(35.29)	
Low	24(70.59)	22(64.51)	
Lymph node metastasis			
Yes	18(52.94)	19(55.88)	0.808
No	16(47.06)	15(44.12)	
Stage of epithelial ovarian cancer			
II	4(11.76)	3(8.82)	0.916
III	15(44.12)	16(47.06)	
IV	15(44.12)	15(44.12)	

### Clinical adverse reactions in group A and group B

The total adverse drug reactions of patients in group A and group B were not significantly different from those in traditional chemotherapy (*P*> 0.05, Table 3).

**Table 3:** Clinical adverse reactions of group A and group B patients

Group	Group A (34)	Group B (34)	P
Leukopenia	3(8.82)	3(8.82)	0.086
Nausea and vomiting	2(5.88)	2(5.88)	1.000
Liver and kidney dysfunction	2(5.88)	1(2.94)	0.555
Allergic reaction	1(2.94)	0(0.00)	0.314
Gastrointestinal tract reaction	2(5.88)	1(2.94)	0.555
Alopecia	3(8.82)	3(8.82)	1.000
Abdominal pain	2(5.88)	2(5.88)	1.000
Total adverse reactions [*n*(%)]	15(44.12)	13(38.24)	0.622

### Clinical effects of group A and group B

Group B (82.36%) had more complete responses or partial response than group A (47.06%) (*P*<0.05, Table 4).

**Table 4:** Clinical efficacy of group A and group B

Group	Group A (34)	Group B (34)	P
CR	5(14.71)	11(32.35)	0.086
PR	10(29.42)	16(47.06)	0.134
SD	10(29.42)	3(8.82)	0.031
PD	9(26.47)	4(11.76)	0.123
Total effective rate of treatment [*n*(%)]	16(47.06)	28(82.36)	0.002

### Comparison of recurrence and metastasis before chemotherapy and after 1 year of follow-up between the two groups

Before chemotherapy, there were 3 cases with rectal metastasis (8.82%), 2 cases with uterine metastasis (5.88%), and 1 case with bladder metastasis (2.94%) in group A, and the total metastasis rate was 17.65% (6/34); before chemotherapy, there were 2 cases with rectal metastasis (5.88%), 4 cases with uterine metastasis (11.76%), and 1 case with uterine metastasis (2.94%) in group B, and the total metastasis rate was 20.59% (7/34). No statistical between-group difference in the metastasis rates before chemotherapy was observed (*P*>0.05).

After 1 year of follow-up, it was found that 17 cases in group A (50.00%) and 9 cases in group B (26.47%) had a recurrence, indicating a significant difference in the recurrence rates after 1 year between the two groups (*P*=0.046).

### Prognosis of patients after chemotherapy

**Fig. 1:**
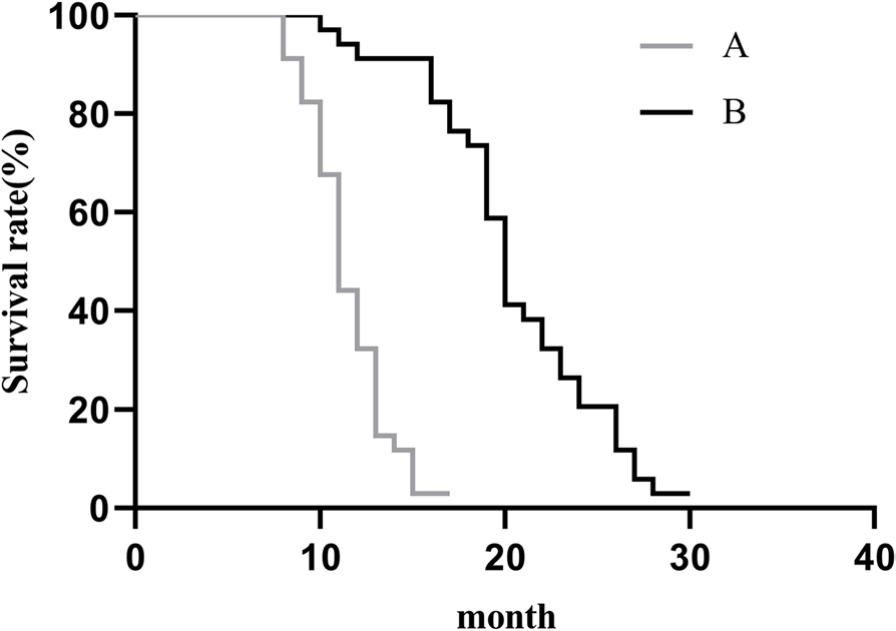
Comparison of median survival of patients in the two groups. The horizontal axis indicated the month, and the vertical axis indicated the survival rate (%); the gray curve indicated the survival curve of group A, and the black curve indicated the survival curve of group B

According to the 1-year survival rate and median survival rate of the two groups of patients, the prognosis was evaluated. The 1-year survival rate was 85.3% (29/34) in group B and 73.5% (25/34) in group A, with no statistically significant difference (*P*>0.05). The median survival time of group B was 20.50 months, significantly higher than that of group A (11.50 months) (*P*<0.05, Fig. 1).

### Comparison of quality of life between Group A and Group B patients

**Fig. 2:**
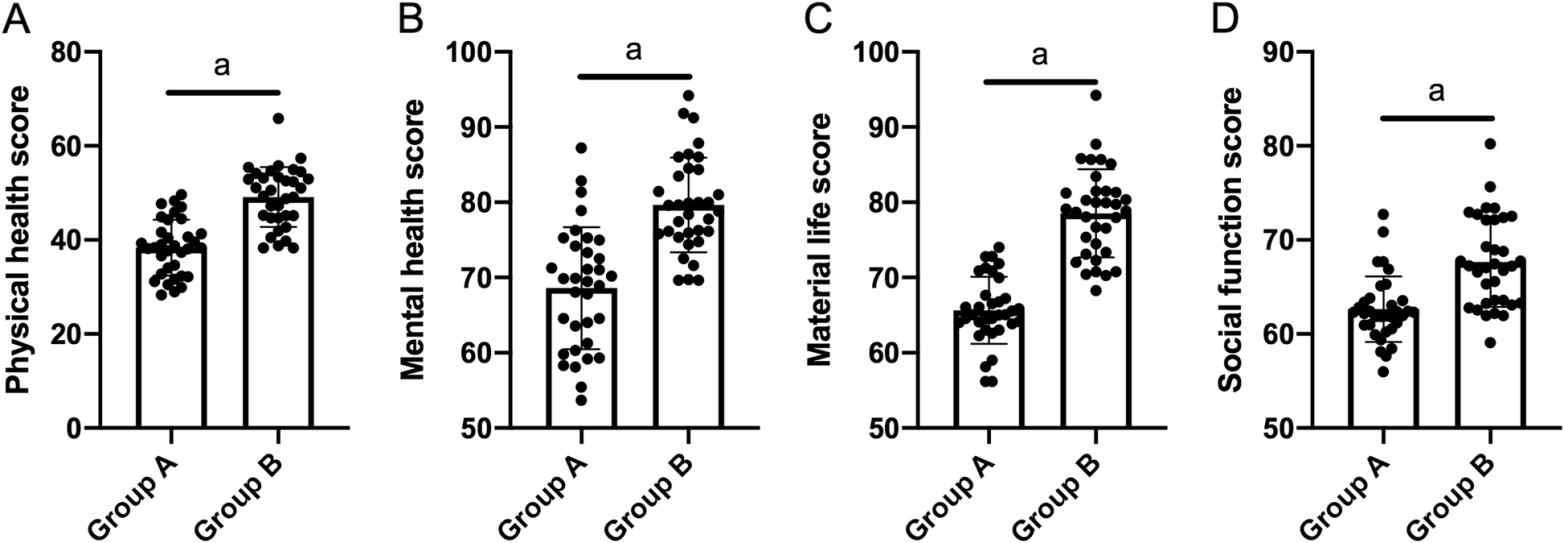
Comparison of quality of life between group A and group B after intervention. The scores of physical health (**A**), mental health (**B**), material life (**C**), and social function (**D**) in group B were significantly higher than those in group A (*P*<0.001)

The QOL-C30 scale scores of physical health, mental health, material life, and social function in group A were (42.00±6.86), (68.15±7.18), (65.31±5.18), and (62.40±4.30), respectively. The QOL-C30 scale scores in group B were (48.60±6.50), (79.58±7.30), (78.23±6.02), and (69.20±5.00), respectively. The quality of life scores of physical health, mental health, material life, and social function of patients in group B were significantly higher than those in group A (*P*<0.001, Table 5, Fig. 2).

**Table 5:** Comparison of quality of life after intervention between group A and group B (x±s)

Group	Group A (34)	Group B (34)	P
Physical health	42.00±6.86	48.60±6.50	<0.001
Mental health	68.15±7.18	79.58±7.30	<0.001
Material life	65.31±5.18	78.23±6.02	<0.001
Social function	62.40±4.30	69.20±5.00	<0.001

### Expression of SDF-1 and CXCR4 in group A and group B before chemotherapy

1. Expression of SDF-1 in group A and group B before chemotherapy

**Fig. 3:**
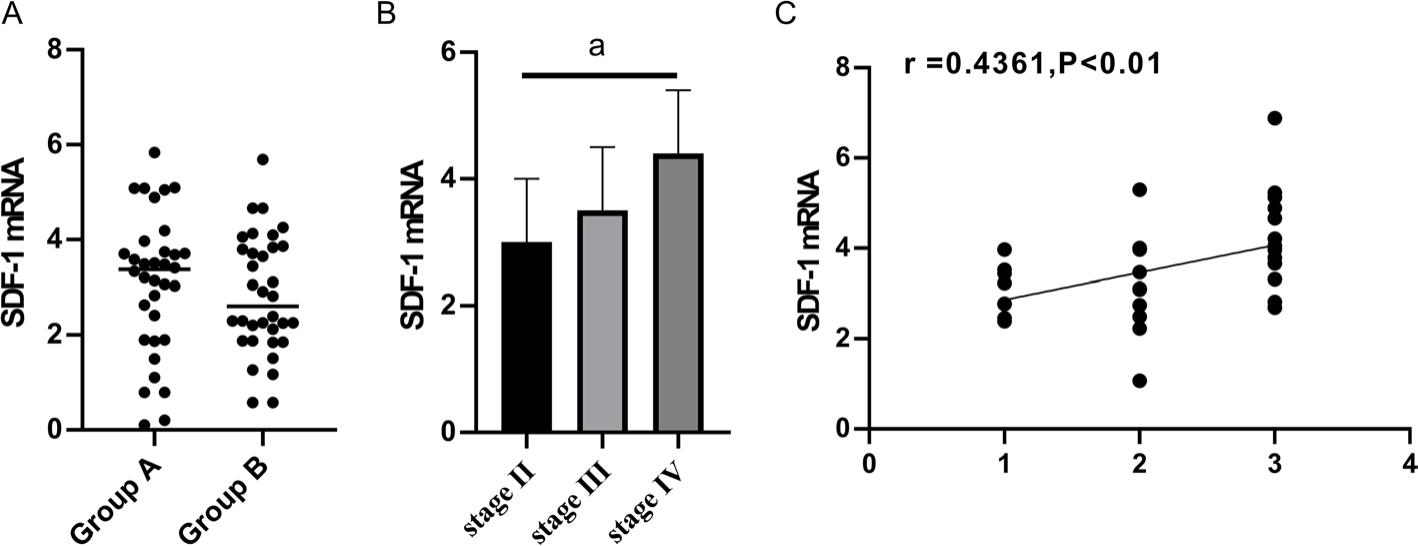
Expression of SDF-1 in group A and group B. **A** Expression level of SDF-1 in group A and group B (*P*>0.05); **B** Expression of CXCR4 increased with the illness of group A patients in stages II, III, and IV, and the relative expression of SDF-1 increased. **C** Spearman correlation analysis showed that the relative expression of SDF-1 was positively correlated with the stage of epithelial ovarian cancer (*P*<0.001)

The expression levels of SDF-1 in group A and group B were (3.20±1.10), and (3.22±1.00) (*P*> 0.05), respectively. The expression level of SDF-1 increased with the illness of group A patients in stages II, III, and IV, and the relative expression level of SDF-1 increased accordingly. The patients at stage II were set as 1, stage III as 2, and stage IV as 3. Spearman correlation analysis between SDF-1 relative expression and different epithelial ovarian cancer stages showed that SDF-1 relative expression was positively correlated with epithelial ovarian cancer stages (*P*<0.001, Fig. 3).

(2) Expression of CXCR4 in group A and group B before chemotherapy

**Fig. 4:**
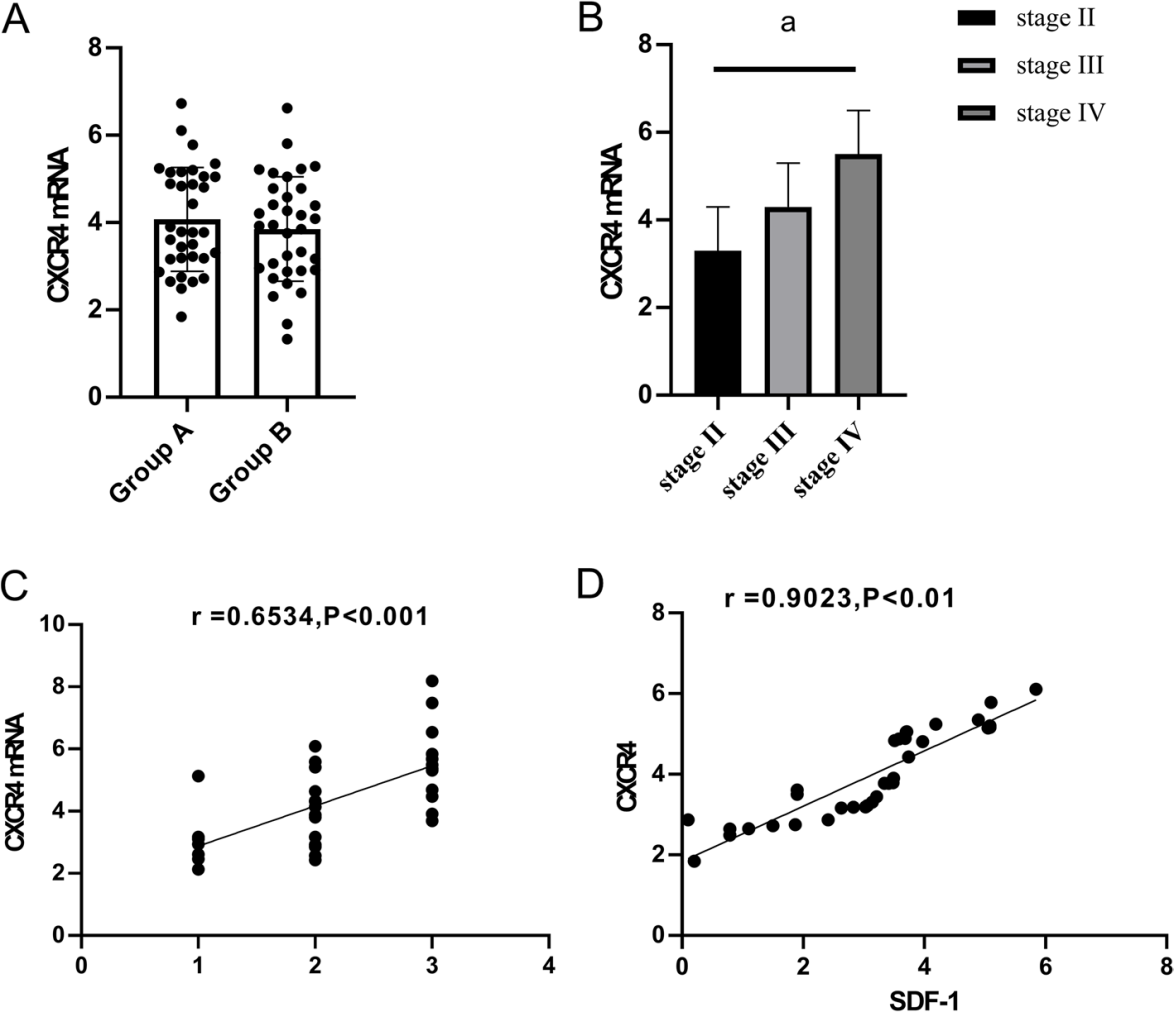
Expression of CXCR4 in group A and group B. **A** Expression level of CXCR4 in group A and group B (*P*>0.05); **B** Expression of CXCR4 increased with the illness of patients in group A in stages II, III, and IV, and the relative expression of CXCR4 increased accordingly; **C** Spearman correlation analysis showed that the relative expression of CXCR4 was positively correlated with epithelial ovarian cancer staging (*P*<0.001). **D** SDF-1 and CXCR4 were positively correlated in epithelial ovarian cancer staging (*P*<0.001)

The expression levels of CXCR4 in group A and group B were (4.10±1.10) and (4.00±1.10), respectively (*P*>0.05). The expression level of CXCR4 increased with the illness of group A patients in stages II, III, and IV, and the relative expression level of CXCR4 increased accordingly. The patients at stage II were set as 1, stage III as 2, and stage IV as 3. Spearman correlation analysis between the relative expression of CXCR4, and different epithelial ovarian cancer stages showed that the relative expression of CXCR4 was positively correlated with epithelial ovarian cancer stages (*P*<0.001). SDF-1 and CXCR4 were positively correlated in epithelial ovarian cancer staging (*P*<0.001, Fig. 4).

### Changes of SDF-1 and CXCR4 after chemotherapy in group A and group B patients

**Fig. 5:**
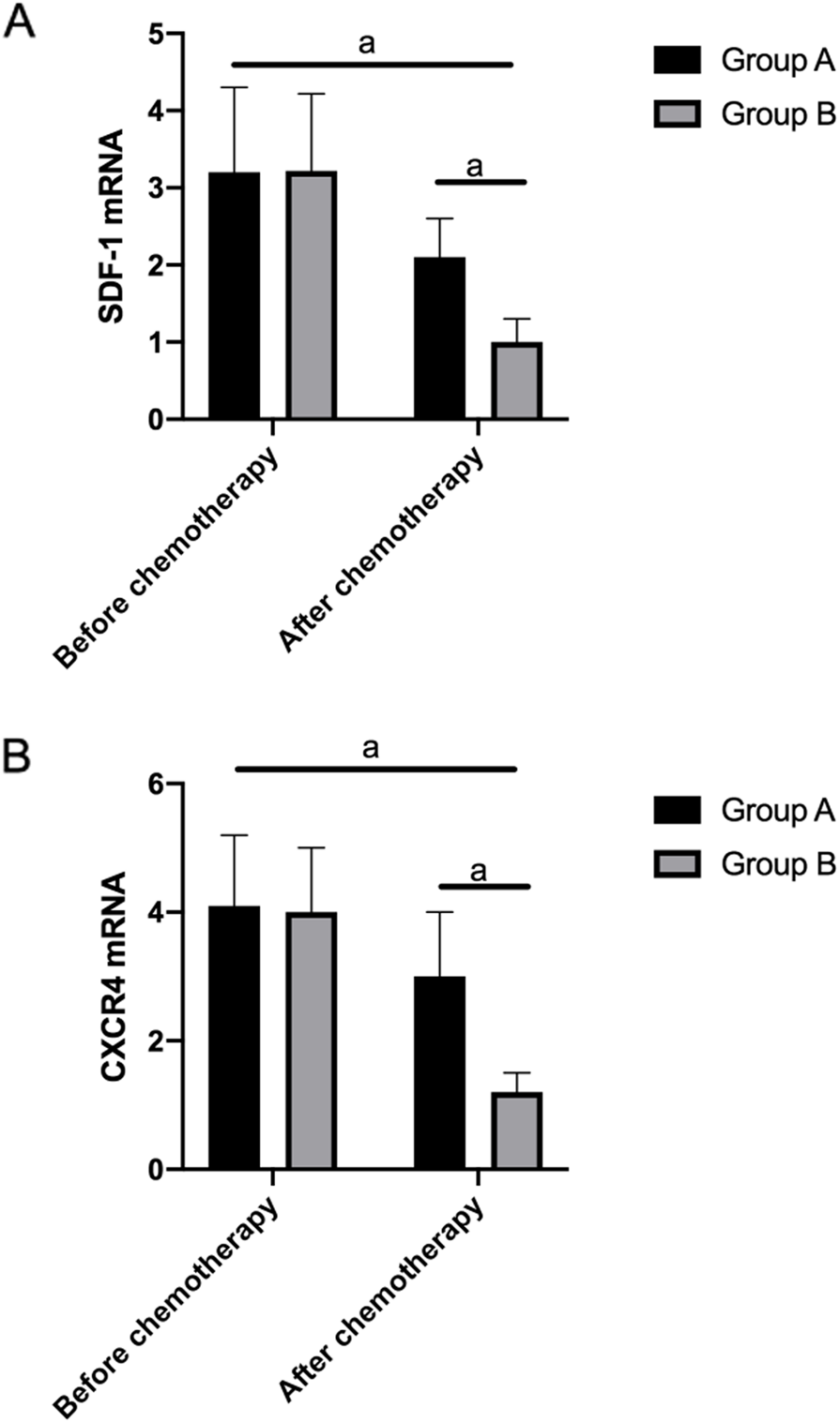
Changes of SDF-1 and CXCR4 in group A and group B after chemotherapy. **A** After chemotherapy, the decrease of SDF-1 in group B was significantly higher than that in group A, a indicates *P*<0.001. **B** After chemotherapy, the decrease of CXCR4 in group B was significantly higher than that in group A, a indicates *P*<0.001

The expression of SDF-1 in group A and group B after treatment were (2.10±0.50) and (1.00±0.30), respectively. CXCR4 expression in group A and group B after treatment was (3.00±1.00) and (1.20±0.30), respectively. The difference of SDF-1 and CXCR4 was statistically significant compared with that before chemotherapy (*P*<0.001), and the downregulation of SDF-1 and CXCR4 expression in group B was significantly higher than that in group A (*P*<0.001, Fig. 5).

### Analysis of prognostic independent risk factors

The overall prognosis of group B was better than group A. Logistic single-factor analysis and multi-factor conditional logistic regression analysis showed that chemotherapy regimen, SDF-1, and CXCR4 were independent risk factors affecting the prognosis of epithelial ovarian cancer (Table 6).

**Table 6:** Multivariate analysis of prognosis and related factors of epithelial ovarian cancer

Factor	*β*	SE	Wald	P	OR	95% CI
Chemotherapy regimen	1.158	2.369	0.000	0.001	1.253	0.430~13.268
Clinical staging	1.103	1.780	0.832	0.024	1.000	0.231~4.251
SDF-1	1.083	1.294	0.759	0.012	1.643	0.172~20.000
CXCR4	2.307	3.852	0.174	0.046	1.736	0.024~2.452

## Discussion

Epithelial ovarian cancer has the highest mortality rate among gynecologic tumors, and most patients are diagnosed as advanced disease [20]. The effective rate of clinical first-line chemotherapy can be 79%; however, the recurrence rate is high. Paclitaxel combined with carboplatin has been used as the first choice for the treatment of epithelial ovarian cancer. Paclitaxel, a natural secondary metabolite isolated and purified from the bark of the Chinese yew (a gymnosperm), has high lipid solubility and low toxic and side effects. It is a new class of anti-microtubule drug, which is mainly effective in anti-cancer by promoting tubulin polymerization and inhibiting its depolymerization, thereby inhibiting tumor cell proliferation and division; and carboplatin, as a second-generation broad-spectrum platinum-based antitumor drug, can interfere with the synthesis process of DNA molecules to produce cytotoxicity and then regulate tumor cell proliferation and apoptosis. Once diagnosed, patients with epithelial ovarian cancer need adjuvant treatment with platinum anticancer drugs. However, many patients are prone to drug resistance to platinum drugs and relapse, which becomes a very difficult clinical problem [21].

In this study, we counted the general clinical data of the two groups of patients, and it was proved that the two groups of patients were comparable. The changes of SDF-1 and CXCR4 before and after treatment, the clinical efficacy, and the safety of treatment plan were observed. First of all, we compared the adverse reactions and clinical effects of patients. The results showed that during chemotherapy, the total adverse drug reactions of patients treated with bevacizumab, paclitaxel, and carboplatin were not significantly different from those of patients treated with traditional chemotherapy. However, the combination had better effect on improving liver and kidney dysfunction, gastrointestinal reaction, and allergic reaction of patients. In addition, the quality of life score of patients treated with bevacizumab, paclitaxel and carboplatin was higher during chemotherapy. The QOL-C30 scale, which could objectively evaluate the current life state of patients from multiple dimensions, was adopted to evaluate the quality of life of patients in the two groups. The study results showed that the scores on patients’ physical health, mental health, material life, and social function were significantly higher in group B than in group A (*P*<0.001), demonstrating that the treatment modality of bevacizumab+paclitaxel+carboplatin had better effect, which could improve the quality of life of patients with epithelial ovarian cancer and help to alleviate the clinical symptoms. In terms of the 1-year survival rate, no significant difference between group B and group A was observed (85.3% (29/34) vs 73.5% (25/34), *P*>0.05). The reasons may be that, after entering the human body, bevacizumab can bind to VEGF and then play the role of inhibiting the proliferation of vascular endothelial cells and neoangiogenesis, thereby achieving the purpose of targeted therapy, also, it has the efficacy of normalizing blood vessels, elevating the delivery of chemotherapy drugs within the tumor tissue, thus better exerting the antitumor effect. Recurrence of epithelial ovarian cancer is seriously deteriorated with little chance of complete cure. Therefore, the treatment of recurrent epithelial ovarian cancer is mainly chemotherapy to control the disease condition, improve the existing clinical symptoms and quality of life of patients, and prolong the survival time of patients [22]. According to the clinical effect after treatment, bevacizumab combined with chemotherapy has better therapeutic effect. Bevacizumab is a monoclonal antibody that inhibits blood vessel growth and can highly bind to vascular endothelial growth factor, thereby reducing the blood supply to the tumor, blocking the supply chain of nutrients and substances needed for tumor growth, and then inhibiting tumor progression. It can be combined with traditional chemotherapy drugs to improve the response rate to treatment and prolong survival of patients with tumors. Clinical studies have shown that the effect of bevacizumab combined with other anticancer drugs in chemotherapy is significantly improved [23]. The chemokine receptor CXCR4 is one of the receptors that has attracted much attention in recent years. The latest study confirmed that CXCR4 interacts with its ligand SDF-1 and is associated with the metastasis of some tumors, and tumor cells with high expression of CXCR4 may, under the chemotactic movement and traction of SDF-1, transfer to some organs that act as a source of ligand production against a concentration gradient, thus forming organ specific metastasis. Therefore, effective regulation of CXCR4 levels in tumor patients is important for stable disease. Then, the expression of SDF-1 and CXCR4 in epithelial breast cancer was detected by qRT-PCR. The relationship of the expression levels of SDF-1 and CXCR4 with the clinicopathological features of severe preeclampsia was analyzed, and Spearman correlation of the relative expression levels of SDF-1 and CXCR4 with different epithelial breast cancer stages was carried out. Based on the analysis results, we assumed that the expression of SDF-1 and CXCR4 in epithelial ovarian cancer patients was positively correlated with the staging of epithelial ovarian cancer. However, there was no significant difference in the expression of SDF-1 and CXCR4 detected by qRT-PCR before chemotherapy. However, after the chemotherapy, the downregulation of SDF-1 and CXCR4 in patients treated with bevacizumab, paclitaxel, and carboplatin was significantly lower than those treated with paclitaxel and carboplatin. At present, although there is no specific study on the effect of bevacizumab combined with chemotherapy on SDF-1 and CXCR4 in epithelial ovarian cancer, there are reports related to SDF-1 and CXCR4 which suggest that the expression of SDF-1 and CXCR4 is upregulated in ovarian cancer patients’ tissues by immunohistochemistry. Furthermore, it was further confirmed by cell experiments that SDF-1/CXCR4 may promote cell proliferation, migration, invasion, and inhibition of apoptosis to make ovarian cancer cell progress, which indicates that the expression changes of SDF-1/CXCR4 are related to ovarian cancer progress. These results were similar to the results of this study and extremely well supported the results of this study [24, 25]. At present, some clinical studies suggest that bevacizumab can improve vascular symbiosis induced by SDF-1α/CXCR4 in patients with glioblastoma [26]. Finally, we analyzed the prognosis of patients after chemotherapy. According to the 1-year survival rate and median survival rate of the two groups of patients, the prognosis was evaluated. The overall prognosis of patients treated with bevacizumab, paclitaxel, and carboplatin was better, and the effect of prolonging survival time was better. In a series of studies on the treatment of recurrent ovarian cancer with bevacizumab combined with chemotherapy, it has been confirmed that the addition of bevacizumab to traditional chemotherapy can effectively prolong the progression-free survival and overall survival of ovarian cancer patients [27]. Logistic single-factor analysis and multi-factor conditional Logistic regression analysis showed that chemotherapy regimen, SDF-1, and CXCR4 were independent risk factors for recurrent epithelial ovarian cancer. However, there has been no previous study on the diagnostic efficacy and predictive value of SDF-1 and CXCR4 expression changes in the placenta tissue of patients with recurrent epithelial ovarian cancer. In this study, SDF-1 and CXCR4 had certain predictive value for the diagnosis and prognosis of recurrent epithelial ovarian cancer.

We confirmed the effect of bevacizumab combined with chemotherapy on SDF-1 and CXCR4 of epithelial ovarian cancer and its prognosis, but there are still some deficiencies in the study. For example, there is no more specific analysis of the specific effects of different chemotherapy courses on the expression changes of SDF-1 and CXCR4. Moreover, there is no exploration on the tolerance of patients to various adverse reactions, which has certain influence on the improvement of research design. Therefore, we will refer to the latest research in real time in the later period and add corresponding research schemes to supplement the defects, so as to continuously improve the research.

## Conclusions

To sum up, the combination of bevacizumab, paclitaxel, and carboplatin affects the expression of related cancer markers SDF-1 and CXCR4. Moreover, the combination of bevacizumab, paclitaxel, and carboplatin has a good improvement on the clinical efficacy, adverse drug reactions, and quality of life of patients with epithelial ovarian cancer.

## Data Availability

The datasets used and/or analyzed during the current study are available from the corresponding author on reasonable request.
